# Strain Decay Monitoring and Analytical Prediction of RC Columns Using Brillouin Optical Technology and Time-Dependent Deterioration Factor

**DOI:** 10.3390/s25030741

**Published:** 2025-01-26

**Authors:** Ittipon Pasityothin, Phromphat Thansirichaisree, Apichat Buatik, Thanongsak Imjai, Radhika Sridhar, Reyes Garcia, Takafumi Noguchi

**Affiliations:** 1 Research Unit of Infrastructure Inspection, Monitoring, Repair and Strengthening, Thammasat School of Engineering, Faculty of Engineering, Thammasat University, Pathumthani 12121, Thailand; ittipon.pasi@dome.tu.ac.th (I.P.); tphromph@engr.tu.ac.th (P.T.); apichat.buat@dome.tu.ac.th (A.B.); 2 School of Engineering and Technology, Walailak University, Nakhon Si Thammarat 80160, Thailand; radhi.kalasridhar@gmail.com; 3 Civil Engineering Stream, School of Engineering, University of Warwick, Coventry CV4 7AL, UK; reyes.garcia@warwick.ac.uk; 4 Building Materials Engineering Laboratory, Department of Architecture, Graduate School of Engineering, The University of Tokyo, Tokyo 113-8654, Japan; takafuminoguchi@ecc-tokyo.ac.jp

**Keywords:** fiber optic sensor, BOTDA, low strength concrete: concrete column, strain decay, structural health monitoring

## Abstract

This study presents a novel approach to the design and assessment of slender reinforced concrete (RC) columns by integrating Brillouin Optical Time Domain Analysis (BOTDA) for real-time, distributed strain monitoring and introducing a “time-dependent deterioration factor” strain decay (η_decay_). Experimental tests on 200 mm × 200 mm RC columns with lengths of 1800 mm and slenderness ratios of 29.4, reinforced with four 12 mm bars, captured strain variations up to 400 microstrain under an axial load of 1200 kN, demonstrate BOTDA’s sensitivity and precision. Unlike conventional strain gauges, BOTDA provided a continuous strain profile along the column height, accurately capturing strain decay with a resolution exceeding 95%, enabling the detection of localized strain reductions often missed by traditional methods. The integration of η_decay_ into ACI 318 and Eurocode 2 models conservatively improved predictions, particularly for specimens tested with long-term testing (720 days), with experimental-to-predicted (E/P) ratios of 1.42 and 1.29, respectively, compared to higher discrepancies in the original codes. The η_decay_ factor accounts for strain reduction along the column height caused by time-dependent effects such as creep, shrinkage, and material degradation, significantly improving the accuracy of axial load capacity predictions. Finite element analysis (FEA) validated these improvements, showing good agreement with experimental data up to the yield load. Post-yield, the modified equations effectively addressed underestimations caused by microcracking, highlighting the necessity of η_decay_ for reliable long-term performance predictions. This research combines advanced BOTDA technology with an innovative η_decay_ framework, addressing long-term structural deterioration and refining design codes. It establishes a robust foundation for integrating time-dependent effects into predictive models, enhancing the resilience, safety, and sustainability of RC structures under real-world conditions.

## 1. Introduction

**Figure 1:**
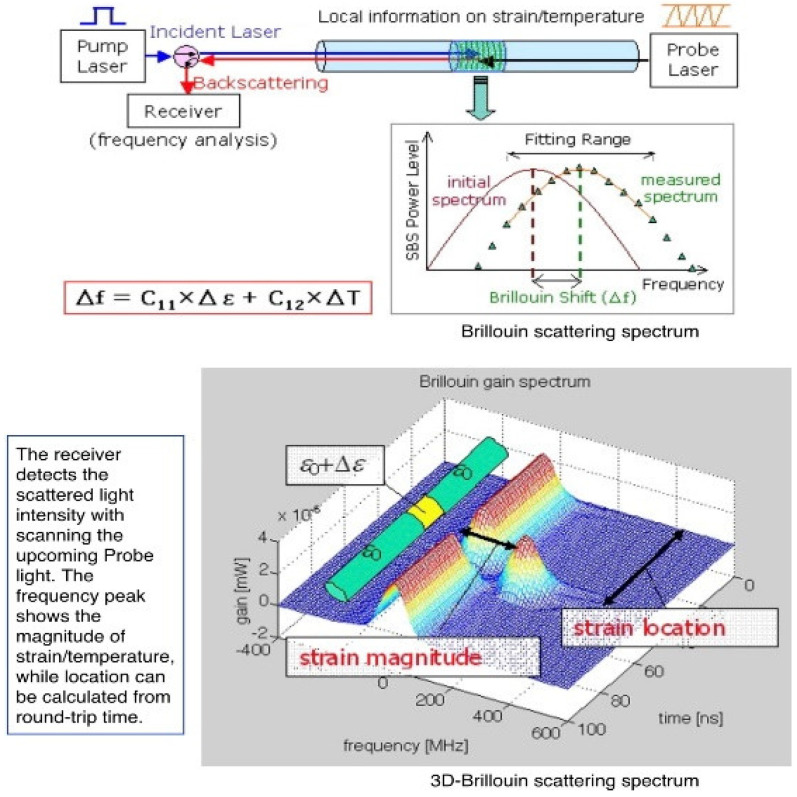
Principle of Brillouin Optical Time Domain Analysis (BOTDA) fiber optic sensor: mechanism of strain and temperature sensing through Brillouin frequency shift analysis [11].

The need for effective structural health monitoring (SHM) in reinforced concrete (RC) structures has grown significantly as the demand for safe, resilient infrastructure increases worldwide [1]. Reinforced concrete columns, essential load-bearing components in many structural systems, are vulnerable to stresses that can lead to cracking, deformation, and potential failure if not monitored accurately. Traditional methods of strain measurement, such as electrical resistance gauges, are limited by their durability and inability to provide comprehensive, distributed data over the entire length of an RC column [2]. Emerging technologies, specifically Distributed Strain and Temperature Sensor (DSTS) specifically Brillouin Optical Time Domain Analysis (BOTDA), offer a promising alternative by allowing for continuous, distributed strain monitoring along a column’s entire length. BOTDA sensors capture data across a wide range of environmental conditions, making them highly suitable for long-term monitoring in RC applications [3,4,5]. The introduction of BOTDA in RC columns provides an innovative solution to traditional monitoring challenges, addressing key limitations while offering improved accuracy and durability [6]. BOTDA is a powerful technique used in fiber optic sensors (FOSs) for measuring strain, temperature, and other parameters along the length of a fiber. It utilizes the interaction between light and acoustic phonons within the fiber to detect changes in the physical conditions of the fibers [7]. The core principle of BOTDA is based on Brillouin scattering, a phenomenon where incident light interacts with the vibrational modes of the fiber, specifically the acoustic phonons. This scattering process leads to the creation of two types of waves: the Stokes wave (lower frequency) and the anti-Stokes wave (higher frequency). The frequency shift between these two waves is highly sensitive to temperature and strain in the fiber with the application of axial loading conditions on the RC columns. The measurement of strain using BOTDA fiber optic sensors leverages Brillouin scattering, where a light pulse interacts with acoustic phonons in the fiber material, causing a frequency shift that is dependent on the local temperature and strain. Figure 1 shows the principle of Brillouin Optical Time Domain Analysis (BOTDA) fiber optic sensor (FOS). When a laser pulse is launched into the optical fiber, it scatters along the fiber, and the backscattered signal is measured [8,9,10]. By analyzing the Brillouin frequency shift (BFS) in this backscattered signal, variations in temperature and strain along the fiber can be inferred. The BFS is sensitive to temperature and strain variations, with specific changes depending on the interaction between the optical fiber’s material properties and external conditions [11]. This shift is localized along the fiber, enabling distributed sensing over long distances. In BOTDA, the light pulse is sent in the time domain, with the scattered signal recorded as a function of time, allowing for detailed spatial information about the FOS under the variational loads [12]. This principle is directly applied in this research to address the challenges of accurately monitoring strain behavior in slender reinforced concrete (RC) columns, which are prone to non-uniform strain distributions due to slenderness effects and long-term material deterioration.

The experimental test was conducted in a controlled environment to minimize temperature fluctuations during testing. The load has been applied to the RC columns at a controlled rate to ensure that strain-induced temperature effects were minimized. Before each test, baseline BFS values were recorded at a reference temperature, and any deviations during testing were adjusted based on these initial readings. BOTDA has been demonstrated to provide high sensitivity and accurate strain measurements in concrete structures, especially in environments where traditional strain gauges and other techniques fall short. The ability to capture distributed strain profiles along the length of the column allows for a more detailed assessment of structural behavior, which is particularly crucial in slender reinforced concrete columns under varying loads and long-term degradation. Despite the slower scanning speed and lower spatial resolution compared to optical frequency domain reflectometry (OFDR), BOTDA has the ability to measure strain at multiple points along the column over large distances, which gives it an advantage in applications where comprehensive strain mapping is required. This provides a global view of strain variations along the entire length of a structural element, which is often more valuable in assessing global structural integrity than localized measurements. Therefore, the utilization of BOTDA in this study is not a disadvantage, but rather a deliberate choice to ensure accurate, long-term, and comprehensive monitoring of RC columns. While BOTDA has demonstrated effectiveness in high-strength concrete applications, its use in low- to medium-strength concrete, such as that found in slender or intermediate RC columns, remains underexplored. Slender RC columns present unique design challenges, primarily due to buckling and stability issues influenced by slenderness ratio and end conditions [13,14,15]. The American Concrete Institute (ACI) code includes provisions for moment magnification factors to account for these slenderness effects; however, conventional models often lack the granularity needed to accurately predict strain behavior in intermediate columns under load [16]. Preliminary results suggest that incorporating the time-dependent deterioration factor in form of the “strain decay” can improve load capacity predictions by up to 10%, aligning well with finite element analysis (FEA) results [17]. This improvement indicates the potential for more accurate and reliable design guidelines for slender RC columns, ultimately enhancing their safety and resilience in diverse structural contexts [18].

Ultrasonic testing has long been a prominent method for evaluating the condition of concrete, as it is sensitive to changes in material properties such as compressive strength and internal cracking. For instance, reference [19] developed an automated ultrasonic inspection system that uses deep learning to analyze the signals from ultrasonic transducers placed on concrete surfaces. Exposure to aggressive environments, such as those containing sulfates, can lead to the degradation of cement-based materials, causing issues like expansive reactions, cracking, and a reduction in compressive strength. Past research [20] proposed the use of an optimized deep learning model for evaluating the compressive strength of cement-based materials exposed to sulfate environments. By using artificial neural networks (ANNs) trained on a large dataset that included factors such as sulfate concentration, exposure time, and temperature, the model could predict the compressive strength degradation with high accuracy. Guo et al. [21] utilized deep learning algorithms, specifically U-Net convolutional neural networks (CNNs), for crack detection in RC structures based on images captured by drones or ground-based cameras. The deep learning model is trained to differentiate between surface cracks caused by environmental factors and those resulting from structural weaknesses. The integration of machine learning and deep learning with traditional diagnostic methods has significantly enhanced the assessment and monitoring of RC structures.

This study addresses the critical gap in monitoring strain and predicting the performance of slender RC columns under axial loads, particularly in low- to medium-strength concrete. Traditional methods, including strain gauges and current ACI 318 [22] and Eurocode 2 [23] guidelines, fail to capture distributed strain behavior and long-term deterioration effects along the column height. To address these challenges, this research investigates the behavior of 18 RC column specimens with variations in slenderness ratios, reinforcement configurations, and fiber optic sensor (FOS) installations. The specimens are grouped into six series with heights of 1.5 m, 1.7 m, and 1.8 m and cross-sectional dimensions of 200 mm × 200 mm and 150 mm × 150 mm. The FOS installations include four types: on main reinforcement, on shear reinforcement (Type B), on the concrete surface (Type C), and near-surface mounted within a groove (Type D). This study integrates Brillouin Optical Time Domain Analysis (BOTDA) for real-time, distributed strain monitoring and introduces a “time-dependent deterioration factor” to account for strain reduction along the column height. This novel approach experimentally validates the strain decay concept and demonstrates its effectiveness in improving ACI 318 and Eurocode 2 predictions, particularly beyond the yield load, enhancing accuracy and reliability. The findings provide a robust framework for incorporating advanced optical sensing technologies and strain decay analysis into current design codes, contributing to safer, more durable RC structures and paving the way for innovative structural health monitoring solutions in civil engineering.

## 2. Experimental Program

### 2.1. Design of Column Specimens

Table 1 illustrates the details of the tested RC columns. The variations in slenderness ratio of the RC columns were considered, along with the different installation methods of FOS throughout the height of the specimens for measuring the strain behavior under the axial compressive load. The experimental programme for this study included 18 RC column specimens grouped into six series, with each series containing three identical specimens. These specimens were cast simultaneously and subjected to testing at different durations: 28 days, 360 days, and 720 days, as detailed in Table 1.

This testing schedule allowed for a comprehensive evaluation of the effects of aging and time-dependent factors such as creep, shrinkage, and material degradation on the performance of RC columns with the significance of the testing duration as follows:

- t = 28 days testing: establishes baseline performance, capturing initial strength and stiffness of RC columns under early-age concrete conditions.
- t = 360 days testing: evaluates intermediate effects of aging, highlighting the influence of creep and shrinkage on strain distribution and structural response.
- t = 720 days testing: assesses long-term performance, validating the “time-dependent deterioration factor” by capturing significant strain reduction and material degradation.

**Figure 2:**
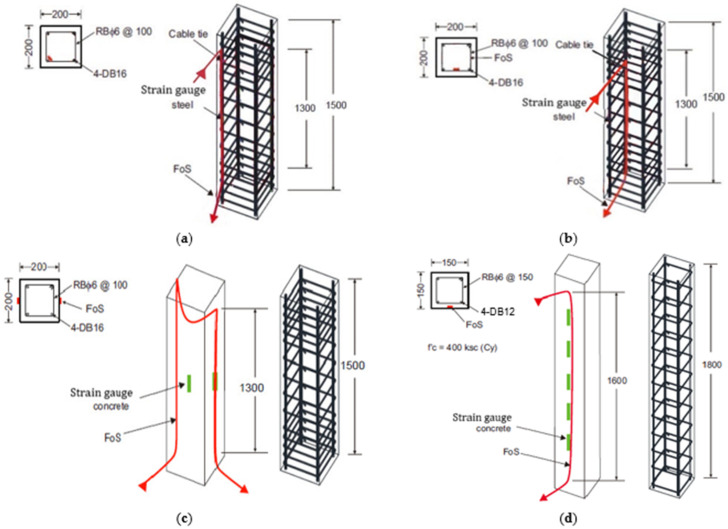
Details of RC columns: (**a**) dimensions of series I with type A FOS; (**b**) dimensions of series II with type B FOS; (**c**) dimensions of series III with type C FOS; (**d**) Dimensions of series VI with type D FOS (units: mm).

The experimental test matrix of the current study consisted of eighteen RC columns with different slenderness ratios and installation methods of BOTDA fiber optic sensors. In this experimental study, strain measurements of RC columns were carried out using both BOTDA fibers and strain gauges. The BOTDA fibers were installed in the RC columns in four different ways, such as (i) FOS placed only on the main reinforcement of the columns (Type A); (ii) FOS placed only on the shear reinforcement (Type B); (iii) FOS placed on the surface of the concrete (Type C); and (iv) grooves induced in the longitudinal direction of the RC column throughout the height and fibers placed onto the groove (near surface mounted—NSM and Type D). The dimensions and reinforcing details of all series of RC column are shown in Figure 2.

The columns had three different total heights of 1.5 m, 1.7 m, and 1.8 m, with cross-sections of 0.2 m × 0.2 m and 0.15 m × 0.15 m. Six different series (I to VI) of testing were performed to study the strain variations of the RC columns through strain gauges and BOTDA FOS. Series I to III had the height of the column as 1.5 m and a cross-sectional area of 200 mm × 200 mm, with 4-DB16 main reinforcements and RBϕ6 mm stirrups at a spacing of 100 mm, as shown in Figure 2a–c. Similarly, series IV and V had the heights of the column as 1.7 m and 1.8 m, respectively, with a cross-sectional area of 200 mm × 200 mm, 4-DB12 main reinforcements, and RBϕ6 mm stirrups at a spacing of 200 mm. The last series of tests was carried out with a cross-section of 150 mm × 150 mm and 1.8 m height to assess the strain’s variational behavior under axial loading conditions, as shown in Figure 2d. The specific installation of strain gauges and FOS to the RC columns prior to casting concrete was precisely carried out to evaluate the behavior of strain variations in the structural system. As discussed, FOSs were installed in the RC columns (Type A, B, C and D) in four different ways, which is also shown in Figure 2a–d. Series I to III had the same column heights (1.5 m) with different FOSs embedded in the system, such as FOS on the main reinforcment, FOS on the shear reinforcement, and FOS on the concrete surface, as shown in Figure 2a–c, respectively. Series IV to VI included the installation of FOSs on the groove, which was induced prior to the placment of FOS on both sides of the columns in the longitudinal direction, as shown in Figure 2d. The optical fibers used for BOTDA measurements were typically placed (shear reinforcement, main reinforcement, concrete surface and groove onto concrete throughout the height) to ensure that they could detect strain concentrations around the reinforcement bars as well as concrete. In the experimental setup, the fibers were placed in positions where strain gradients were expected, ensuring that strain concentrations around the reinforcement and concrete were captured accurately through the data acquisition system.

### 2.2. Materials Used

#### 2.2.1. Concrete

**Figure 3:**
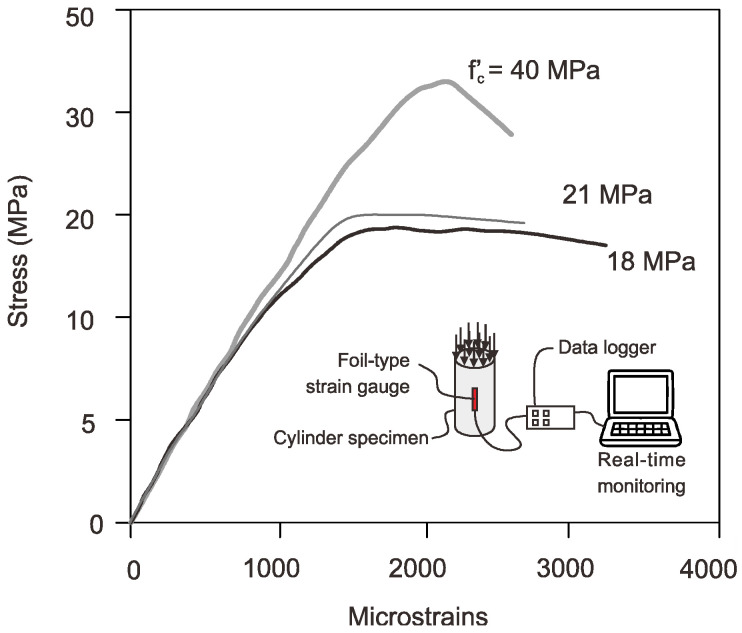
Stress–strain relationship of concrete obtained by foil type strain gauges (SGs).

In accordance with the mix designs, as per ACI 211.1 [24], three concrete mixtures were made to cast different series of RC columns. For every combination, ordinary Portland cement (OPC) Type I was utilized to produce concrete with the desired strength. Mixtures m1, m2, and m3 were produced with water-to-cement (*w*/*c*) ratios of 0.45, 0.42, and 0.36, respectively, to obtain the target compressive strength. Natural coarse and fine aggregates, in accordance with ACI 211.1 [24], were used for all mix proportions. For all mixtures, the desired slump was intended to obtain compressive strength levels of 85 mm and 95 mm. To make the mixtures more workable, 3% to 5% of superplasticizer (SP) by volume was added to obtain a target compressive strengths of 18, 24, and 40 MPa concrete. Table 2 illustrates the concrete mix proportions per cubic meter. Standard concrete cylinders 300 mm in height and 150 mm in diameter were used to determine the compressive strength and strain values. Notably, all concrete bodies were water-cured, and plastic sheets were placed over the concrete to stop the concrete from hydrating too quickly. Strain gauges of the foil type were adhered to the cylinders to measure the strains. The stress–strain plot of a concrete elastic of a cylinder is shown in Figure 3. Note that only the foil-type (SG) was attached to the cylinder concrete specimen to capture the stress–strain curves, as the fiber optic sensor (FOS) was only applicable to specimens with span lengths over 1200 mm due to the high velocity of the pulse signal [11,12].

From the stress–strain plot, it is revealed that the low strength concretes had higher strain values compared to higher compressive strength concrete. The decrease in strain value for 40 MPa compressive strength concrete was due to the brittleness effect of concrete specimens. The low-strength concrete may have been the cause of the increased strains in “M18” mixes, which range between 3100 and 3300 microstrains, furthermore increasing the deformability of the concrete. The best slope of the cylinder’s stress–strain curve was used to validate the results in numerical analysis. It is important to note that the hysteresis loop was designed with the optimal slope. When the load was removed, the concrete did not regain its former shape, causing hysteresis. The mechanical properties of the concrete are tabulated in Table 3.

#### 2.2.2. Reinforcement Bars

The Young’s modulus and tensile strength of the reinforcement bars were obtained from direct tension tests. The extensometer had a gauge length of 100 mm. Although slippage could still happen during the test, the ends of each steel specimen were securely held to prevent it. The average value of the best slope of the load–displacement plots of all tested specimens with the same diameter was used to determine the modulus of elasticity of the steel bars. The yield (*fy*) and ultimate (*fu*) strengths of steel were calculated from the average of three specimens for the main reinforcement as 398 MPa and 520 MPa, respectively. Similarly, the calculated yield (*fu*) and ultimate strength (*fu*) of the shear reinforcement were 370 MPa and 480 MPa, respectively.

### 2.3. Test Setup and Instrumentation

The loading test setup and instrumentation of the RC columns to measure the strain behavior through BOTDA and conventional strain gauges are discussed in this section. The strain behavior was measured through the innovative BOTDA measuring principle to evaluate the effects of different slenderness ratios of the RC columns.

#### 2.3.1. Loading Arrangement and Procedure

**Figure 4:**
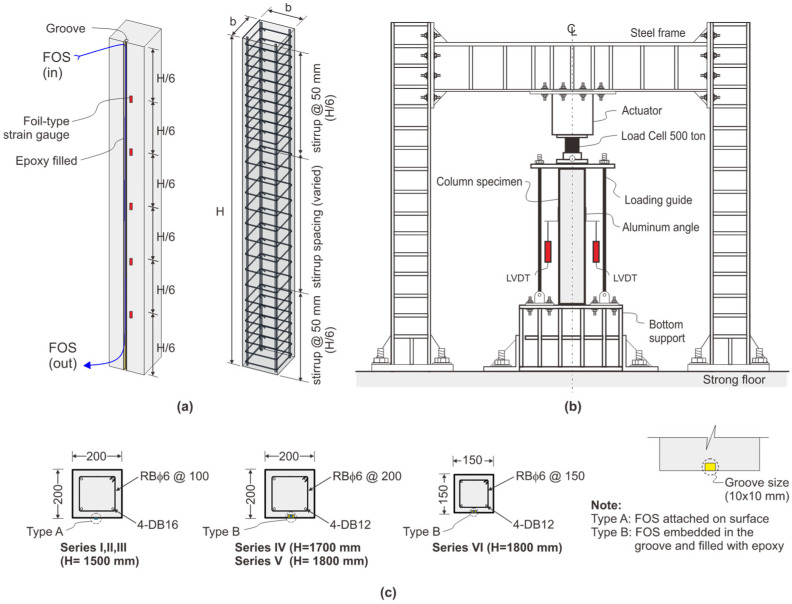
Schematic view of experimental test setup. (**a**) Reinforcement details and installation of strain gauge and FOS. (**b**) Test setup of RC column under axial loading conditions. (**c**) Cross-section and installation type of sensors for all series.

A universal testing machine with the capacity of 5000 kN was used to evaluate the performance of RC columns under compressive loading conditions. All the RC columns were tested under a servo-controlled hydraulic machine to ensure that the RC column was under controlled loading conditions, accurately simulating the applied forces and ensuring performance under various stress scenarios. As per the hierarchy of the experimental program (Table 1), in all series (Series I to VI), three RC columns were cast-tested at the ages of the 28 days, 360 days, and 720 days to investigate the time-dependent strain variational behavior through conventional strain gauges and BOTDA sensors under the axial loading conditions. The overall deformation was measured using linearly variable displacement transducers (LVDTs). A schematic view of experimental setup is shown in Figure 4.

**Figure 5:**
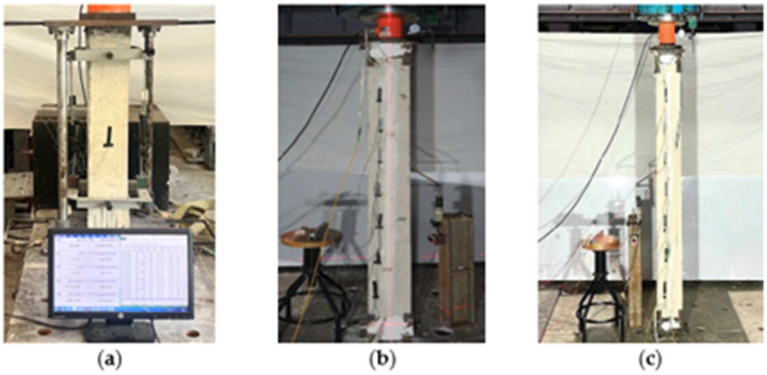
Loading arrangement of RC column. (**a**) RC column with strain and FOS measurements. (**b**) Typical view of columns with LVDTs. (**c**) RC column under axial test.

BOTDA fiber optic sensors and foil type strain gauges were used to measure the strain under the applied load. The placment of the FOS was fastened to the RC column in four different ways, as discussed in Section 2.2. Figure 4a shows the typical installation of FOS onto the induced groove of RC columns near surface mounted region along with the attachment of the foil type strain gauge to measure the strain variations. Figure 4b shows the test setup of RC columns under axial loading conditions, which were placed between the bottom support and the loading cell actuator for the specific application of load through the hydraulic machine. For all series of experiments, FOS and stain gauges were placed before the casting of the concrete for types A and B using epoxy and, in the cases of types C and D, the FOS was placed after the casting of the concrete on the concrete surface and groove, respectively, as shown in Figure 4c. In an axial compression test, a uniform strain distribution was assumed along the length of the RC column in the experiments conducted for this project. The estimated theoretical strains in columns for any tiny compressive strains can be used to determine the strain distribution in the elastic range. It was calculated that steel bars and concrete had a perfect bond and would not slide across one another. Even though the concrete’s Young’s modulus changes with strain, calculations can be performed using concrete considered as an elastic body since it is possible to assume a constant Young’s modulus for low strain values. The strain measurements of 10 kN to 70 kN loading increments and column distance towards its longitudinal direction of RC column through BOTDA fiber optic sensors were calculated. The distributions of strain from the strain gauges and FOS were plotted for the three different loads based on the ultimate and failure loads of the RC columns. A typical view of the experimental test setup of the RC column is shown in Figure 5.

#### 2.3.2. Deflection Measurement

Figure 4b shows the test setup arrangement of the RC column with top and bottom platens attached and LVDTs in all four directions to evaluate the displacement of the column. The top platen of the loading machine had a roller system for the application of uniform load. The BOTDA readings took seven to eight minutes to complete and were taken at every load increment. Three specimens were cast and tested for each series of experimental programs. For all experiments, the BOTDA base reading was taken approximately between the cracking load and ultimate load. The load was increased by a 100 με interval, which was equivalent to 50 kN. Fluctuations of loading were observed, as this was a displacement-controlled mode in which hydraulics frequency self-adjusted to retain constant displacements. The load was increased up to the equivalent strain of 450 με and then decreased to a 100 με step. For concrete to be within an elastic range for the cube strength, the approximate strain value is 450 με. Because a column or a pile often runs within this range, which is necessary for a serviceability limit state, the modest strain value within an elastic range is appropriate for this experiment.

#### 2.3.3. Strain Measurement and Installation

**Figure 6:**
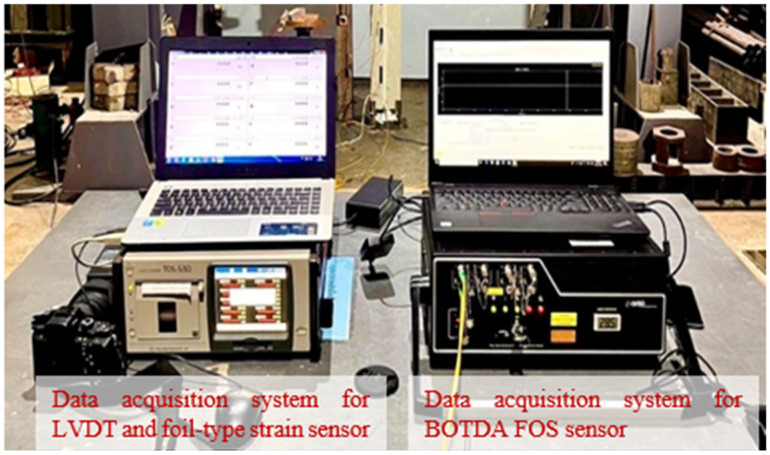
Typical view of data acquisition system for LVDT, as well as the foil-type strain sensor and BOTDA fiber optic sensor strain connection measuring system used in the study.

Optical fibers can be used as sensors to measure strain, optical loss, temperature, pressure, and other parameters. Smart structures have been incorporating optical fiber sensing as the primary tool in their monitoring system. Brillouin Optical Time Domain Analysis (BOTDA) fiber was used in this experimental work and delivered continuous strain measurement based on back-scattered light characteristics. The Brillouin scattering frequency rose proportionately to the strain exerted at the scattering region and shifted by 11 GHz from the incident light in a single-mode fiber. The working principle of BOTDA fiber optic sensor (FOS) strain measurement was discussed in Section 1. A typical view of BOTDA fiber optic sensor strain variations measured from the RC columns is shown in Figure 6. The experimental setup for this study utilized a Distributed Strain and Temperature Sensor (DSTS) system, manufactured by OZ Company, to measure strain distribution along the length of reinforced concrete (RC) columns under axial loading conditions. The key setup parameters included a Central Brillouin Frequency of 10,850 MHz, a Spatial Step (Data Discretization) of 0.04 m, a Pulse Width of 5 ns, an Average Number (Signal Averaging) of 10,000, an Input Range of 200 mV, and the use of LSHZ Grade 652D optical fibers. The Brillouin Frequency Shift (BFS) was extracted using the standard Local Correlation Function (LCF) method, ensuring robust detection and characterization of distributed strain along the optical fibers. To minimize environmental influences, all tests were conducted under controlled laboratory conditions, with baseline BFS values recorded prior to applying axial loads. This setup enabled precise and repeatable measurements, providing a reliable foundation for analyzing strain variations in the RC columns.

In this experimental work, by connecting strain variations to an optical fiber and considering the change in refractive index and the speed of an acoustic wave moving through the fiber, BOTDA calculates the strain variations under the application of axial loading conditions. Their interactions are related to the electro strictive effect of fiber, which tends to compress materials when an electrical field is present. When a pump pulse wave and a counter-propagating continuous probe wave travel from two ends of the optical fiber and come into contact at different locations along the fiber, electrostriction modulates the density and, consequently, the refractive index of the optical fiber, as shown in Figure 6. The acoustic wave energizes the waves of the pump reflected light power at a sound speed in the fiber material. The acoustic wave in this instance is known as stimulated Brillouin scattering.

**Figure 7:**
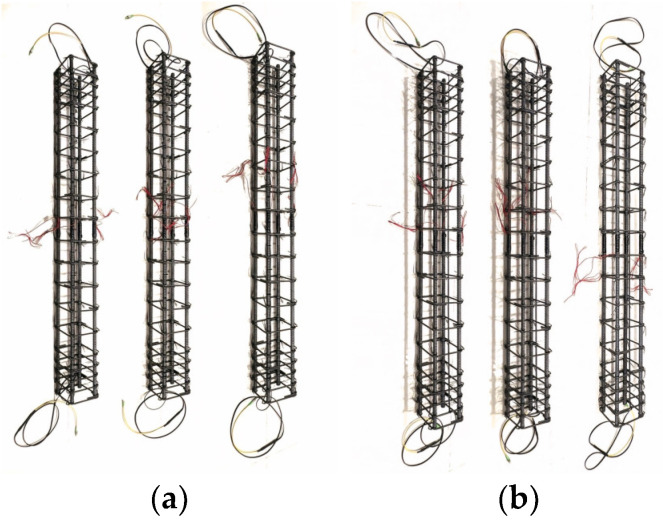
Installation of strain sensors on reinforcement cage of RC column and FOS in Series I (**a**) and Series II and III (**b**).

Steel rebars were used to connect the longitudinal bars and shear links. Figure 7 displays images of both columns with strain gauges and FOS. Changes in strain within the concrete columns were tracked using strain gauges. To achieve high strain measurement accuracy, the strain gauge installation procedure needed to be extremely precise. The installation of a strain gauge is depicted in Figure 7.

To stop water from seeping through the concrete paste, the strain gauge was adhered to the rebar host’s immaculately polished surface using silicone glue. Ten strain gauges were mounted on the main steel reinforcement of the column, spaced about 100 mm apart for a column 1.8 m in height. One steel bar was inserted with three strain gauges at the midsection and at both ends, and three rebars had one strain gauge at the midsection of the column in the case of an RC column 1800 mm in height, as shown in Figure 7. To minimize the effects of temperature on the strain measurements, experiments were conducted under controlled environmental conditions, which helped to reduce fluctuations that could otherwise interfere with the precision of the BOTDA measurements.

Some challenges can arise during the installation of the BOTDA fibers, such as the correct integration and alignment of the optical fibers within the concrete, as well as the embedment of the fibers, without compromising the structural integrity or the bond between the reinforcement and the concrete. To mitigate for these potential issues, a careful installation and alignment procedure is needed (e.g., routing the fibers along predetermined paths), as well as careful casting and consolidation procedures for the concrete mix. Figure 7 shows the typical attachment of an optical fiber to a steel rebar. To track the amount of applied strain, one end of the optical fiber is attached to the BOTDA FOS. The optic fiber is re-clamped onto the table after the necessary pre-strain has been reached. To secure the optical fiber to the host rebar, epoxy glue is used at intervals of 10 to 15 cm. In the case of a two-point fixation or clamped method, the installation procedure is same as the glue method. Before the pile cage is installed, an optical fiber is clamped onto a steel cage in a pile at two opposing ends.

## 3. Results and Discussion

This section examines the experimental behavior of RC columns under axial loading, focusing on strain distribution, load capacity, and displacement behavior. Section 3.1 analyzes the ultimate load capacity and failure modes, highlighting the influence of slenderness and reinforcement on structural integrity. Section 3.2 explores load–displacement relationships, detailing stiffness degradation and ductility variations across specimens tested at 28, 360, and 720 days.

### 3.1. Ultimate Capacity and Failure Behaviors

Table 4 shows the experimental test results and performance indices of RC columns. The energy dissipation (*z*), ultimate load (*P_u_*), yield load (*P_y_*), first cracking load (*P_cr_*), maximum deflection (Δ*_max_*), yield deflections (Δ*_y_*), stiffness (*S_e_*), and ductility (*µ*) at failure and the ultimate load of all tested RC columns were obtained from the experimental tests. At the start of the loading operation, the damage pattern of the RC column specimens under axial compressive loadings resembled that of the traditional RC column specimen in the case of columns (C1 to C9). But when the load grew closer to the specimens, the ultimate carrying capacity was enhanced with the increase in stiffness [25]. In this study, the columns were cast with low-to-medium concrete strengths (18–40 MPa), as these are representative of the typical concrete strength widely used in construction applications of residential and commercial buildings throughout Southeast Asia. As such, further research should investigate the applicability of the approach proposed in this study to high-strength concrete or even ultra-high-performance concrete (UHPC). Such concretes exhibit different characteristics, including greater brittleness, higher modulus of elasticity, and potential for reduced ductility and energy dissipation capacity. Large sections of the concrete cover spalling and significant longitudinal rebar local buckling occurred for the RC columns with 1.5 m height under ultimate stress conditions. From the test results, it has been observed that the ultimate load (*P_u_*) was greater for the C6-100 RC column with the reinforcement ratio of 2.0% (ρ*_f_*). There was no significant crushing of the concrete columns at 1.8 m height with a cross-sectional area of 150 mm × 150 mm.

Later, compared to RC column specimens, there was more noticeable longitudinal rebar bucking and concrete spalling in the case of RC columns without prestressing. Failure patterns of RC columns for different reinforcement ratios and slenderness ratios are shown in Section 4. for all series. Additionally, longitudinal compression rebars were shown to exhibit local buckling. On the other hand, longitudinal cracks developed initially in specimens with the increase in yield load, and the yielding of longitudinal tension rebars was the last form of failure. When compared to the specimens with heights of 1.8 m, the longitudinal compression reinforcement local buckling was more noticeable. There were no discernible differences between the failure modes of the prestressed RC columns and conventional RC columns.

### 3.2. Load vs. Displacement Curves

**Figure 8:**
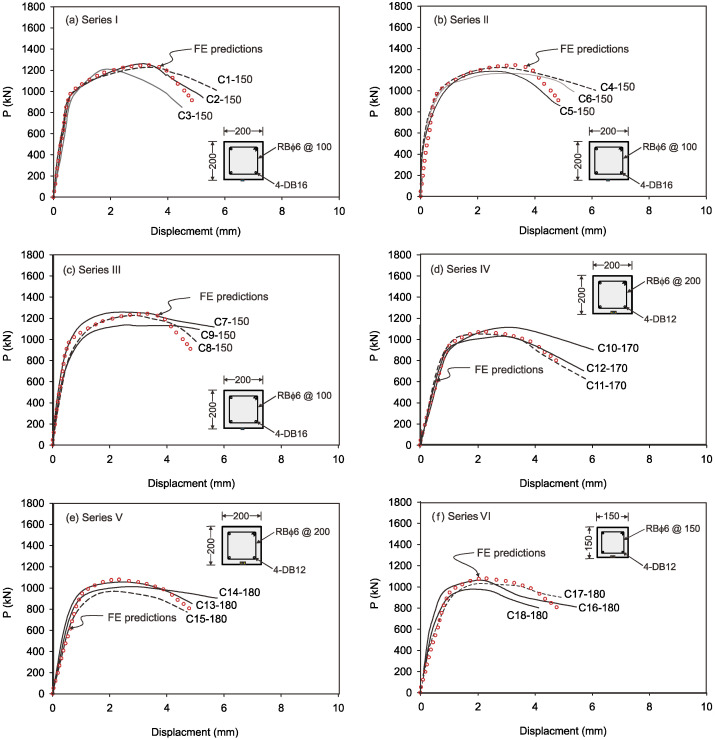
Comparison of experimental and numerical values for load versus deflection of RC columns.

The load versus deflection of RC columns under axial compression behavior for all series and for three times (28, 360, and 720 days) is shown in Figure 8. From the load deflection curves, the main points of the curve under axial compressive behavior from series I to III, in the case of a similar height of 1.5 m and a cross-sectional area of 200 mm × 200 mm, are depicted in Figure 8a–c, which show the initial concrete cracking, concrete cover spalling, longitudinal bar yielding, ultimate load point, and failure load point, respectively. The axial displacement associated with the peak load is indicated by (Δ*_max_*). Table 4 displays these points’ displacement values, such as the displacement at yield point (Δ_y_), displacement at ultimate (Δ_u_), and displacement at failure load point (Δ_f_). From the tested experimental values (Table 4) and load versus deflection (Figure 8), it was observed that the ultimate load (P_u_), yield load (P_y_), and first cracking load (P_cr_) decreased with the increase in the number of days from 28 to 720 days.

The decreases in the ultimate load for the RC column specimen C1-150 were about 9.2% and 17.2% at 360 and 720 days, respectively, compared to the ultimate load at 28 days of testing. Similar observations were made for all series of RC columns under axial compression loading conditions, which may be attributed to the time-dependent deformation shown in Figure 8a–f. It is evident that the increase in the number of days decreased the deflection capacity of the RC columns, due to which the load-carrying capacity and the ductility factor at the ultimate and failure loads were also decreased.

The longitudinal bars gave way after the initial concrete cracking for these axially stressed RC column specimens. At the same time, as displacement increased, concrete coverings gradually spalled. The restricting effect of RC columns was then fully demonstrated for the column with height of 1.5 m, before the specimens reached their peak loads [26]. Figure 8d,e show the load versus deflection behavior of RC columns with heights of 1.7 m under axial load for series IV and V. Furthermore, in the case of series IV, the displacement with respect to the loads RC column specimens rapidly decreased following the peak load. However, compared to the curves of the RC columns in series I to III, the loads in the descending portion of the RC column curves in series IV and a respectable number of peaks fell more slowly.

It can be inferred that, due to more efficient confinement, RC columns with the 1.5 m height can achieve superior structural performance when compared to RC columns with 1.7 m height when the volume ratio of reinforcement is well-designed. Similarly, in series VI, a prestressed concrete RC column was cast and tested under axial compression load, the load versus deflection curve of which is shown in Figure 8f. It was shown that the highly prestressed concrete column specimen did not fully develop the confinement effect of the reinforcement [27]. The concrete covers were all delaminated around the peak loads when all specimens were compared. All of the curves for the prestressed concrete columns resembled the RC columns in terms of shape and tendency to vary with the displacement. Additionally, it was shown that the prestressed concrete columns had a far smaller restricting impact than the axial-loaded columns compared to series I to V.

### 3.3. Strain Measurement from BOTDA FOS and Strain Gauge

**Figure 9:**
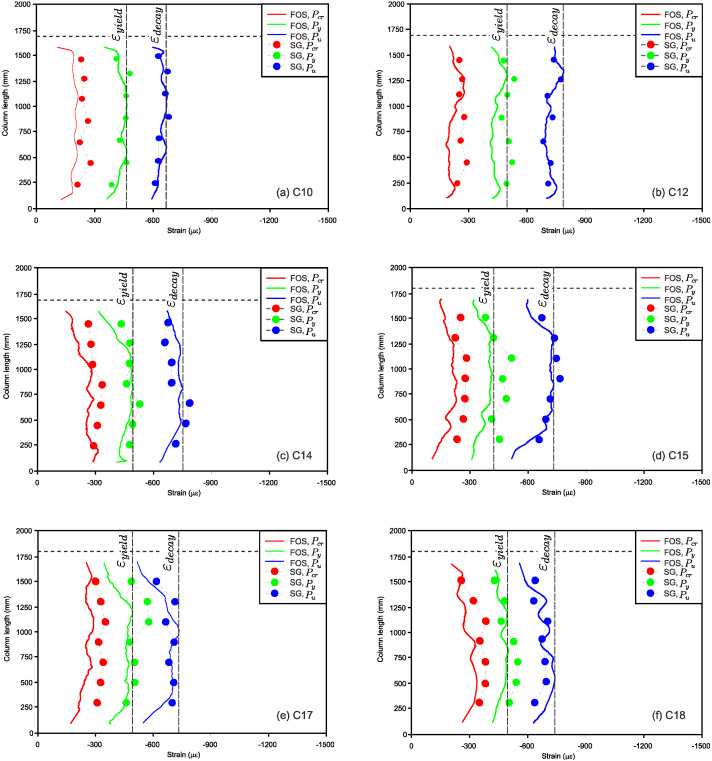
Column height versus strain vales from FOS and conventional strain gauges at 720 days. (**a**) C10−Series IV, (**b**) C10−Series IV, (**c**) C12−Series V, (**d**) C15−Series V, (**e**) C17−Series VI, and (**f**) C18−Series VI.

Figure 9 shows the typical BOTDA FOS and strain gauge readings along the specimen’s centerline under three different axial loads of RC columns based on the ultimate and yield loads.

From the experimental test, strain values were taken from FOS at first cracking load (P_cr_), yield load (P_y_), and ultimate load (P_u_) to study the behavior of RC columns under time-dependent deformation. The strain variational behavior was evaluated under the axial compression load for all series of columns at the age of 720 days. The strain values at the yield point ($\varepsilon_{y}$) and maxiumum strain values ($\varepsilon_{d\;}$) were caluculated from Figure 9a–f to evaluate the ultimate load for the proposed equations through ACI 318 [22] and Eurocode 2 [23] in order to capture the “strain decay” ($\eta_{d\;}$) parameter. A moving average of ten points was used to raise the effective sensor gauge length throughout the coulmn to account for possible fiber position misalignment. This was judged to be suitable for the examined square column dimensions inside the concrete with the FOS attachment on the main reinforcement, shear reinforcement, concrete surface, and groove. Figure 9 shows the idealized strain estimates based on basic strain gauge measurement, omitting slip in the cross section and placing the neutral axis 100 mm from the one end of the column. The elastic strain distribution was a piecewise linear line that resembles a semi-parabolic curve as a result of the RC column being subjected to axial load.

The overall shape agreement between the measured strain distributions from the FOS and the strain gauge was quite similar. Measurements from the resistance-based strain gauges fixed on the surface of the columns as well as the reinforcement bars closely matched the FOS strains at different sites [28]. In contrast to conventional strain gauges, which only provide information at specific places, FOS measurements have the advantage of providing information regarding the continuous strain distribution along the specimen’s length. Local strain concentrations around the reinforcement bars, spatial variations of the height of the RC column in conformance with the buckling due to the slenderness ratio, heterogeneity in the concrete material, or local variations in the optical fiber path could all be causes of the local variations in the FOS strain in this large test specimen.

The fact that peaks arose at the same places in all FOS fibers with different amplitudes, however, leads the scientists to infer that much of the differences were related to the periodic change in the slab depth and headed studs. There was no discernible damage or strain variation information from the FOS at the cracking load level for columns from series I to III or the yield load level for columns from series IV to VI, as shown in Figure 9a,f. At this load level, however, the strain profile along the height of the column was clearly visible from both the FOS and conventional strain gauges. Figure 9b–d display the FOS and strain gauge (concrete and reinforced steel bars) results for the RC column specimens C13, C15, and C17, respectively. The different strain instants were separated for the same load level (that is, the measured strain and strain decay at 10% level) in order to examine the impact of axial loading on the FOS observations. All of the FOS and conventional strain gauge results coincided at this yield and ultimate load levels. This indicates that all FOSs provided accurate readings for the present load level under axial loading condition, and that there was no additional damage under the loading for the current load level of the yield point. Figure 12b shows the development of crack patterns under the loading level of the cracking point. The steady rise in strain in the FOS from top to bottom along the height of the column signifies rising curvature brought on by a rising axial load. Comparing the ultimate load under axial condition reveals the asymmetric behavior of the column, which experienced greater maximum compression under loading conditions. More precisely, due to the concentration of deformation close to the slot, the highest compression strain along the height of the columns was around 300 micro strain at a loading level of 200 kN.

At the load level of cracking, with the yield and ultimate load levels for series I to III and series IV to VI, a slight degree of energy dissipation was released by the addition of axial load onto the column. The cracking pattern at the load level of cracking load (300 kN) is shown in Figure 12b. However, when the column was under compression, the fissures along the sides of the column were evident through the cracking pattern figure at the load level of 700 kN. These crack positions support the likelihood that they were present in the FOS at the 300 kN load threshold. Because the deformation concentrated closer to the slot while the other end of the column was under strain, the cracks all around the top portion of the columns were quite invisible at this loading level. The FOS results from the figures provide additional confirmation of this variation in the deformation mechanism [29]. The deformation was localized close to the top side of the column, whereas the strain on the other side of the column gradually grew or declined.

Figures 12–17 show the FOS results of the RC columns at a load level of 300 kN. At this load level, energy loss under axial loading was more noticeable from the cracking pattern of Figure 13b. The figure showed a greater number of cracks and wider cracks in comparison to the earlier load levels, which further suggests an increasing degree of damage [30]. The elevated degree of RC column damage can be interpreted using the strain distributions from the existing FOS data. The FOS cable buckled due to localized concrete damage at the maximum loading level. As was mentioned in the preceding section, this buckling, along with probable debonding and slippage between the reinforced bars and the cable, caused a net positive strain at the damaged area. In the meantime, the other side of the column also showed damage, with a concentration of greater strains occurring over the height of the column.

## 4. Numerical Study

### 4.1. Geometry, FE Model and Boundary Conditions

**Figure 10:**
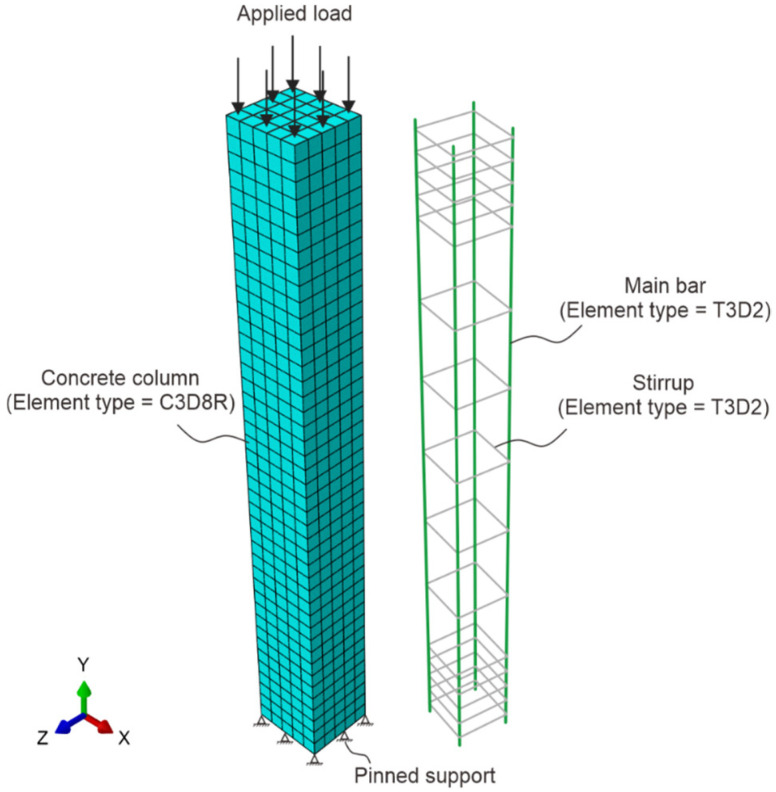
Modeling of RC columns in Abaqus^®^; C3D8R-3D solid element for concrete modeling; and T3D2-3D truss element for main and shear reinforcements.

To gain deeper insight into the performance on RC columns, non-linear finite element analysis (FEA) was carried out using Abaqus^®^ software (version 6.14). These investigations considered both geometric and material nonlinearities. The concrete of the RC column was modeled using 8-node linear brick elements, as shown in Figure 10. The concrete was modeled using a modified second-order integration technique and C3D8R solid elements. The main and shear reinforcements were modeled using 2-node linear 3D truss elements, as shown in Figure 10. The reinforcement steel bars were modeled using an element type T3D2. An 8 mm mesh size was chosen after an optimization of mesh sensitivity was performed in Abaqus^®^ [31]. The boundary conditions of the RC columns were kept pinned at one end and free at another end as shown in Figure 10.

This process was carried out automatically by the software to minimize errors that could result from distortions throughout the analysis. To simulate the actual experimental test, an axial load was applied to the RC column. The number of steps at which the numerical analysis was to cease was established using the maximum load ascertained by the tests carried out during the experimental test. According to earlier research [32], this technique is sufficiently precise to measure the behavior of RC columns, such as deflections and buckling. The test findings from the concrete and steel material property results were used to perform the modeling of the RC column. Numerical analyses of the RC columns were carried out to evaluate the serviceability behavior under axial loading conditions for different slenderness ratios of the column.

### 4.2. Concrete Damage Plascticity Model and Applied Loading

**Figure 11:**
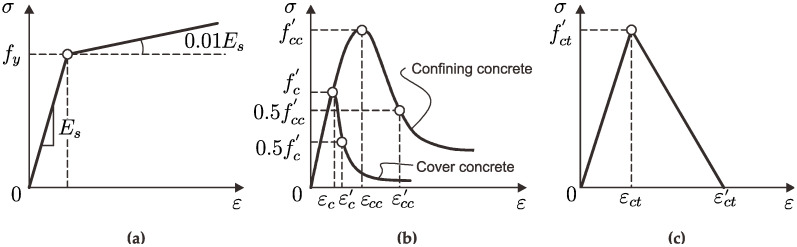
Constitutive relationships used in this numerical study. (**a**) Stress–strain of reinforcement steel. (**b**) Compressive stress–strain relationship. (**c**) Tensile stress–strain relationship.

Considering the material properties from the experimental test results described in Section 2.1, the analyses employed a concrete damaged plasticity (CDP) model since it has been demonstrated to be accurate enough to replicate the axial load behavior of RC column structures. Using the built-in concrete damage plasticity (CDP) model in Abaqus^®^, the behavior of concrete with compressive strengths of 18 MPa, 24 MPa, and 40 MPa was simulated based on the specimen respective cross-sections along with the reinforcements. In this experimental study, a constitutive stress–strain relationship of reinforcement was chosen as a bilinear model to evaluate the non-linear behavior of RC columns under axial loading conditions [33]. The constitutive stress–strain relationship of reinforcement is shown in Figure 11a. The constitutive relationships of compressed plain concrete shown in Figure 11b and the confined concrete in the core region led us to select the stress–strain model proposed by [31] in consideration of the confining effect of the stirrup on the columns. The FE modeling carried out in this study assumed a perfect bond between the concrete and the reinforcement. Whilst this assumption was sufficiently accurate to simulate the experimental results presented here, real structures can experience some bond slip between the concrete and the reinforcing bars, thus leading to localized strain variations and to less uniform strain distributions. However, BODTA measurements can detect whether bond degradation occurs as localized strain changes, which can be subsequently incorporated into the FE modeling.

The stress–strain curve of concrete under tension is shown in Figure 11c. The uniaxial stress–strain models of RC columns were modeled in this finite element analysis. The value of stress (0.5*f’_c_*) in the stress–strain curve under axial compression indicates the point at which the concrete began to deviate from linear elastic behavior. The development of microcracks in the concrete matrix, a frequent feature at this site, led to a nonlinear stress–strain relationship. After that, as the pressure and tension grew, the concrete gradually lost its rigidity until it was close to breaking. The plastic zone was represented by strain softening beyond the ultimate stress and stress hardening up to the maximum stress (*f’_c_*). Under uniaxial tension, the stress–strain relationship was linear-elastic until the failure stress (*f’_t_*_0_), which is when microcracking began. Crack formation in the compression zone when subjected to axial loading was indicated by a softening stress–strain response that resulted in strain localization in the concrete structure beyond the failure stress. For the numerical analysis (Abaqus), creep and shrinkage effects were modeled as part of the material properties through time-dependent material models, which simulated these phenomena. The deterioration factor (e.g., damage indices DAMGEC and DAMGET) would be influenced by these time-dependent behaviors, where the stiffness and bearing capacity degradation would occur more rapidly because of creep and shrinkage over time.

### 4.3. Comparison of the FE Predictions and Experimental Results

**Figure 12:**
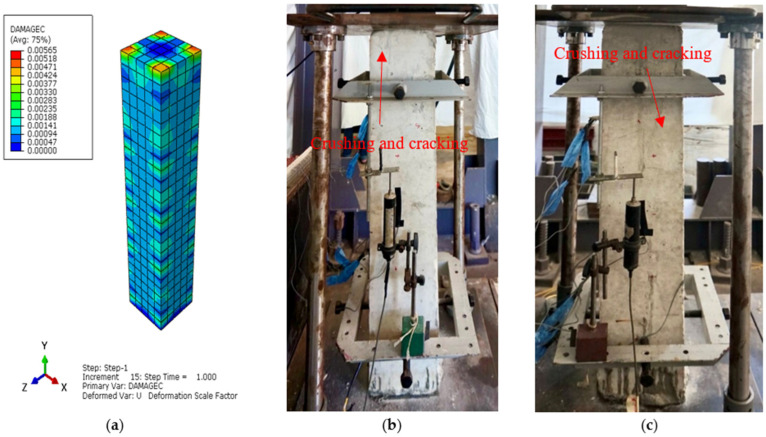
Damage index analysis of RC column—C1 series I. (**a**) DAMGEC. (**b**) Cracking pattern of C1-150 column at cracking load. (**c**) Cracking pattern of C1-150 column at ultimate load.

**Figure 13:**
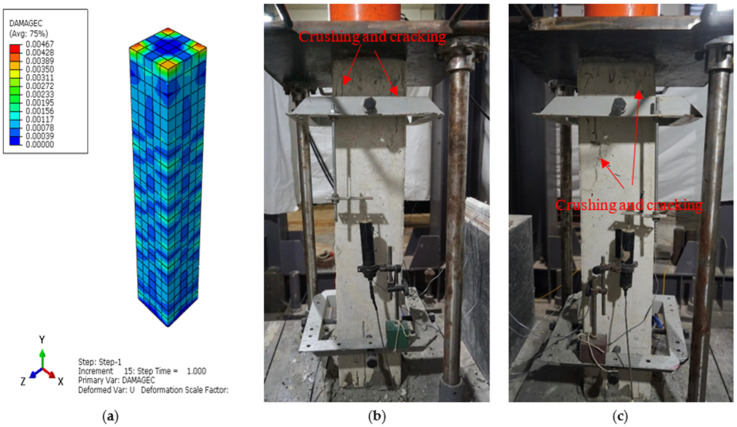
Damage index analysis of RC column—C5 series II. (**a**) DAMGEC. (**b**) Cracking pattern of C5-100 column at ultimate load. (**c**) Cracking pattern of C5-100 column at failure load.

**Figure 14:**
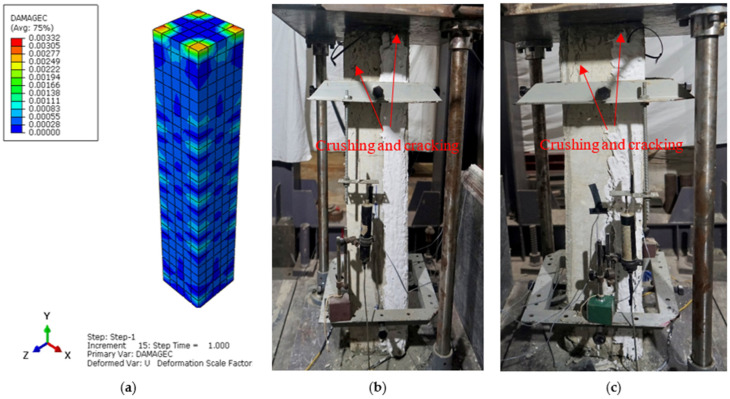
Damage index analysis of RC column—C9 series III. (**a**) DAMGEC. (**b**) Cracking pattern of C9-100 column at cracking load. (**c**) Cracking pattern of C9-100 column at failure load.

**Figure 15:**
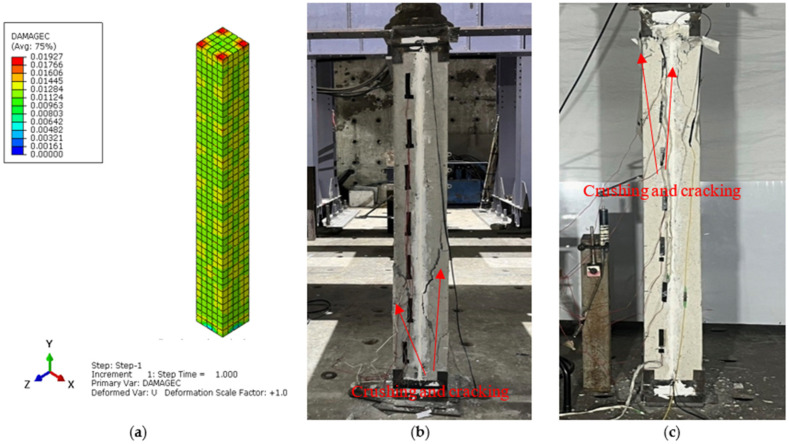
Damage index analysis of RC column—C12 series IV. (**a**) DAMGEC. (**b**) Cracking pattern of C12-170 column at ultimate load. (**c**) Cracking pattern of C12-170 column at failure load.

**Figure 16:**
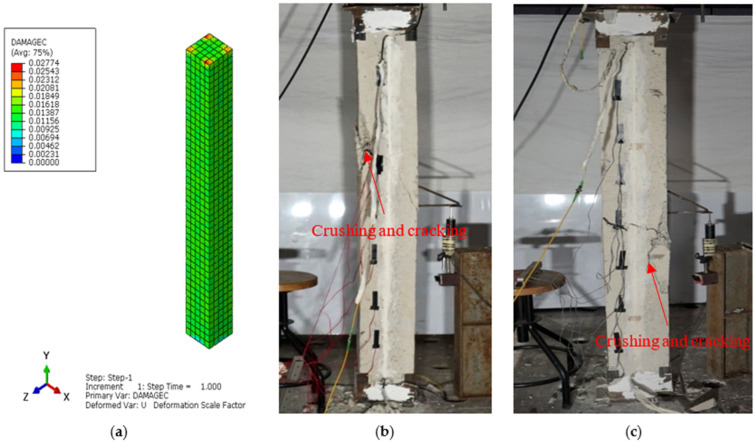
Damage index analysis of RC column—C15 series V. (**a**) DAMGEC. (**b**) Cracking pattern of C15-170 column at cracking load. (**c**) Cracking pattern of C15-170 column at failure load.

**Figure 17:**
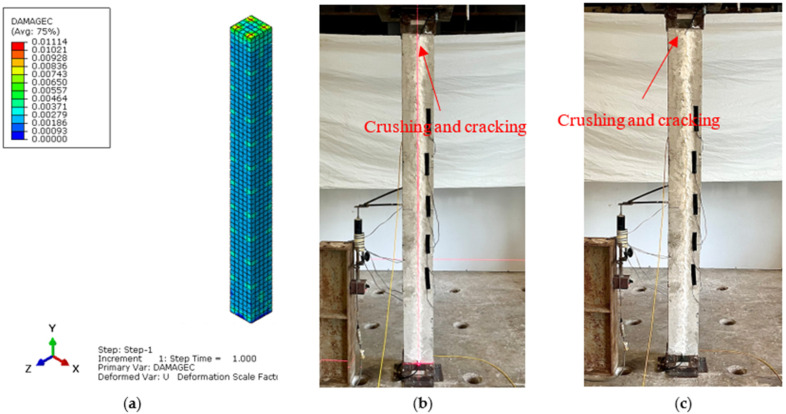
Damage index analysis of RC column—C18 series VI. (**a**) DAMGEC (**b**) Cracking pattern of C18-180 column at ultimate load. (**c**) Cracking pattern of C18-180 column at failure load.

The main aim of the finite element (FE) analysis was to predict the experimental load and strain behavior of RC columns under axial compression loading conditions. The FE model was validated in Abaqus^®^ for all series of RC columns by considering the material properties from the experimental tests. Compressive damage (DAMGEC) and tensile damage (DAMGET) are the two damage index definitions used in Abaqus^®^. The experimental and numerical load versus displacement curves are shown in Figure 8, Section 3.2, for all series of RC columns. The overall patterns of the curves from the numerical simulations closely matched those of the experiments. The load versus deflection curves under axial loading conditions were complete in shape for shorter columns, in contrast to columns over 1.7 m in height. Furthermore, columns under axial loads for series I had greater ductility and bearing capacity than those of the columns in series V. This phenomenon, which is linked to the detrimental effect of the displacement on the lowering of the column height, shows that columns exhibit higher ultimate load and ductility capacity [34]. After attaining the maximum bearing capacity, the curves showed a downward trend, indicating a loss of stiffness. Table 4 illustrates a comparison of the performance indices of RC columns from the experimental study, which aligns well with the numercial load versus deflection parameters. Nevertheless, the load–displacement curves derived from the numerical simulation did not exhibit the same stiffness phenomena as the experimental results. This might be because the numerical simulation simplified the bond-slip between the concrete and reinforcement as well as the tension of the reinforcement itself. For the specimens subjected to the same loading technique, as illustrated in Figure 8b, the ultimate load decreased as the height of the column increased, but the bearing capacity increased [35]. Figure 12, Figure 13, Figure 14, Figure 15, Figure 16 and Figure 17 show the compression damage index analysis and cracking patterns under different axial loading conditions for all series of RC columns (series I to VI).

The ultimate load of column C-9 dropped more quickly than columns C-3 and C-5. This response demonstrates that there was acceptable axial compression, because increasing the height of the column lowered the ductility and energy dissipation capability of the specimens. The strain from the numerical analysis, according to Von Mises criteria, and the displacement of the RC column in “x” and “y” directions were also in good aregement with the experimental values. The Abaqus^®^ analysis came to an end after a certain number of steps, as indicated by the ultimate load, which corresponded to the ultimate load.

The maximum strain under the axial compressive load was approximately 0.0045, which is consistent with Von Mises yield stress criterion stress values, and the maximum obtained deflections in both the “x” and “y” directions of the same column were 0.59 mm and 0.42 mm. Similarly, for all series of RC column specimens, the experimental test results ere validated through finite element analyses to cross-check the perfromance behavior of columns under axial loading conditions. From the results, it was revealed that the FEA predictions were in good agreement with the experimental results, and henceforth, Abaqus^®^ simulation is rationally recommended to assess the performance behavior of RC columns under axial loading conditions. All specimens showed decreases in the bearing capacity degradation coefficient, as displacement increased under axial compression loading conditions, as shown in Figure 12a (DAMGEC), for columns 1.5 m in height (C1 to C9). The bearing capacity of the specimens deteriorated more quickly when the slenderness ratio of the column increased, while the loading techniques remained constant. Furthermore, RC columns with slenderness ratios of 17.3 had a major negative impact on the deterioration of the bearing capacity, which led to the failure of the specimens, as shown in Figure 12b. As illustrated in Figure 13c and Figure 14b, the axial compression caused a decrease in bearing capacity; however, the effect was imperceptible. The average stiffness deteriorated more quickly during the second loading phase, as discussed in Section 3.3.

The stiffness gradually decreased as the displacement increased for the RC columns which belonged to series IV and series V. With the deterioration of bearing capacity, the stiffness degraded more quickly with an increase in the axial load beyond the ultimate loading capacity of the columns, and the presence of cracks and yielding of steel sped up the stiffness degradation during the loading phase from the initial condition to the final stage. Figure 15b and Figure 16c illustrate how the damage index of specimens changed as displacement increased, based on failure criteria as well as the load versus deflection curves from Figure 8d,e. The damage indexes of specimens under axial loadings, with the exception of columns with slenderness ratios of 41.6 are illustrated in Figure 15 and Figure 16. The maximum deflection was 3.72 mm when the simulated columns failed under axial compressive stresses. Furthermore, at the final displacement, RC column C-17, belonging to series V, had deflection values of 1.52 mm and 1.55 mm at maximum and failure conditions, respectively, indicating a versatile failure pattern at the top surface, as shown in Figure 17b. But under the given axial loading from the experimental values, the damage indexes of the RC columns with 29.4 slenderness ratios were lower than those of the columns of series III and VI. It is evident that all damaged RC column sepcimens reached the failure load more quickly than the yield loading condition.

Additionally, the RC columns with slenderness ratios of 41.6 and lower displacement had greater damage than columns 1.5 m in height, as shown in Figure 17c. Nevertheless, under axial compression loadings, all columns belonging to series I to III had lower damage indexes than columns C16, C17, and C18.

Building on insights from the experimental and numerical studies, the following section introduces modifications to ACI 318 and Eurocode 2 equations to enhance their applicability to slender RC columns. The findings highlight the critical role of the time-dependent deterioration factor in addressing strain reduction along the column’s height, which significantly impacts axial capacity predictions. Current code equations often neglect these effects, leading to inaccuracies, especially in post-yield scenarios. By integrating strain decay and long-term deterioration parameters, the proposed modifications provide more reliable and accurate predictions, aligning design standards with the observed performance of RC columns under real-world conditions.

## 5. Modified ACI and Eurocode Equations for Predicting Column Strength

### 5.1. ACI 318 Equation

The ACI 318 code [22] provides a widely accepted equation to calculate the axial capacity of RC columns under concentric loading. The equation is given by Equation (1):

(1) $$P_{n}=0.85\;f_{c\;}^{\prime }A_{g\;+\;}A_{s}f_{y}$$

where $P_{n}$ is the nominal axial load capacity, ∅ is the strength reduction factor (typically 0.65 for compression members), $f_{c\;}^{\prime }$ is the concrete compressive strength, $A_{g\;}$ is the gross cross-sectional area of the column, $A_{s}$ is the cross-sectional area of longitudinal reinforcement, and $f_{y}$ is the yield strength of reinforcement steel.

This equation assumes uniform strain distribution along the height of the column and is primarily calibrated for normal-strength concrete (i.e., compressive strength $f_{c\;}^{\prime }>$25 MPa) under concentric axial loading. However, for low-strength concrete (LSC, $f_{c\;}^{\prime }\leq \;$25 MPa), the equation often overestimates the capacity, particularly for slender columns, due to its inability to account for non-uniform strain distributions and second-order effects.

For slender columns, lateral displacements and P×∆ effects influence the axial capacity. To address these, the ACI 318 code introduces a moment magnification factor $(M_{m}$) to account for slenderness effects. The adjusted Equation (1) becomes Equation (2):

(2) $$P_{n}=\emptyset \left(0.85\;f_{c\;}^{\prime }A_{g+}A_{s}f_{y}\right)\times \;\;\frac{1}{1-\frac{Pu∆}{EI}}$$

where Δ is the lateral displacement due to slenderness, *EI* is the flexural stiffness of the column, and *P_u_* is the applied axial load.

While this adjustment improves predictions for slender columns, it does not consider the observed decay in strain along the column height, which becomes significant in LSC columns due to their lower stiffness and pronounced non-linear behavior. This strain decay is critical in slender columns, where the effective strain decreases strain with height, leading to overestimating capacities when using the unadjusted equation.

### 5.2. Eurocode 2 Equation

The Eurocode 2 [23] approach differs significantly by considering a broader set of factors, including effective stiffness, imperfections, and slenderness effects. The nominal axial capacity (*N_Rd_*) for a column under concentric axial load is given in Equation (3):

(3) $$N_{Rd}=\alpha \times A_{C}f_{cd}+A_{s}f_{yd}$$

where α is the reduction factor for long-term effects and column stability, $A_{C}$ is the area of the concrete cross-section, $f_{cd}$ is the design compressive strength of the concrete ($f_{cd}=\frac{f_{ck}}{\gamma_{c}}$), $A_{s}$ is the area of reinforcement, and $f_{yd}$ is the design compressive strength of the concrete ($f_{yd}=\frac{f_{y}}{\gamma_{s}}$).

For slender columns, Eurocode 2 includes additional provisions to account for second-order effects by considering imperfections and introducing an effective length factor. The design load is adjusted using the effective stiffness of the columns, accounting for creep and cracking effects. The critical load (*N_cr_*) is calculated in Equation (4).

(4) $$N_{cr}=\frac{\pi^{2\;}{EI}_{eff}}{L_{eff}^{2}}$$

where ${EI}_{eff}$ is the effective flexural stiffness, including concrete cracking and creep, and $L_{eff}$ is the effective length of the column.

Eurocode 2 provides a more detailed approach than ACI 318, especially for slender and low-strength concrete columns. However, it similarly assumes a uniform strain distribution, which may not be valid for LSC with significant strain decay due to the formation of large deformations as well as microcracks under axial compression.

### 5.3. Proposed Modifications to the ACI and Eurocode 2 Equations

**Figure 18:**
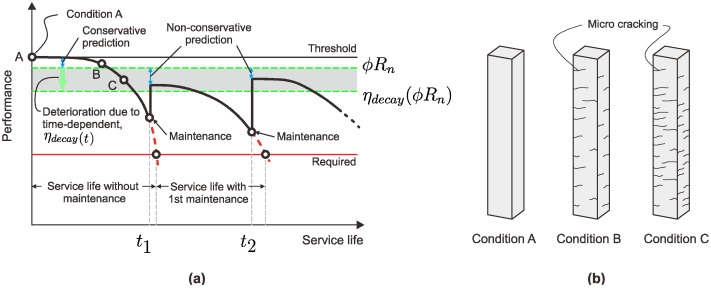
Performance of service life predictions of RC columns through ACI 318 and Eurocode 2: (**a**) life cycles of RC elements and (**b**) decay in performance due to time-dependent deterioration effect.

The ACI 318 [22] and Eurocode 2 [23] eqations have been extensively validated for normal-strength concrete (NSC), but their applicability to low-strength concrete (LSC) columns remains limited. Both approaches assume uniform strain distribution along the column height, which is often invalid for LSC due to its reduced stiffness, higher strain variability, and susceptibility to micro-cracking. These characteristics make LSC columns particularly sensitive to slenderness effects, second-order deformations, and lateral displacements. The strain decay ${(\eta }_{decay}$) was calculated for all the RC columns by considering the values at yield as well as ultimate strain points to validate the conservative prediction of the increase in service life through the FOS. Figure 18 shows the performance of service life predictions of RC columns through ACI 318 and Eurocode 2.

According to the service life prediction with the help of ACI 318 and Eurocode 2, this research proposes ACI 318 and Eurocode 2 equations modified from the strain decay values to understand the condition of the structure after 20 to 30 years of its service life, as shown in Figure 18a. Initially, the threshold load level was evaluated through the experimental investigation, and furthermore, conservative predictions were made with Equations (1) and (3). Later, the same was validated through the explicit Abaqus model to ascertain the strain values under axial load as well as the load versus defection criteria. Conservative predictions were made from the equations given in Equations (1) and (3) according to the total strain decay from the strains with time-dependent parameters, like creep, shrinkage, and microcrack strains (Figure 18b). These parameters were considered in the present study through the FOS to predict the serive life maintenance in order to overcome deterioration due to environmental factors, long-term time-dependent deformations, and structural integrity of the system.

The ACI 318 [22] equation for axial capacity (Equation (1)) lacks provisions for strain non-uniformity. While slenderness is addressed using a moment magnification factor, $M_{m}$, the equation’s accuracy diminishes as slenderness ratios increase, particularly when combined with the low stiffness of LSC. Similarly, Eurocode 2 [23] improves upon slenderness modeling through critical load calculations and effective stiffness adjustments, but still relies on uniform strain assumptions. These limitations lead to significant overestimations of axial capacity, especially in LSC columns with slenderness ratios exceeding 30. Existing code equations, such as those in ACI 318 and Eurocode 2, are designed for normal-strength concrete and primarily assume uniform strain distribution. They inadequately address the complexities introduced by time-dependent deterioration effects, which significantly influence strain behavior, especially in slender RC columns. Current predictions fail to account for strain decay caused by long-term factors such as creep, shrinkage, and microcracking, leading to inaccuracies in post-yield and long-term performance assessments.

To bridge this gap, this research proposes the integration of TDD into current code predictions. By quantifying and incorporating strain decay into predictive models, the modified equations align more closely with experimental observations, ensuring safer and more reliable designs. Addressing this gap is essential for advancing design standards, enhancing the durability of RC structures, and providing more accurate service life predictions in real-world applications. Recent advancements in BOTDA provide an oppurtunity to address these limitations. BOTDA enables real-time monitoring of strain distribution along the entire height of a column, capturing variations in strain caused by slenderness, lateral displacements, and material properties. A key insight from BOTDA data is the concept of “strain decay”, which represents the gradual reduction in strain along the column height. This behavior, particularly pronounced in LSC columns, underscores the non-uniformity of strain distribution that traditional models fail to capture.

In this study, “strain decay” ($\eta_{d}$) is introduced as a correction factor to improve the predictive accuracy of both ACI 318 and Eurocode 2 equations. The $\varepsilon_{d}$ factor is derived from BOTDA measurements and quantifies strain reduction, typically ranging from 5% to 15%, depending on column geometry, material properties, and loading conditions. By intergrating this factor, axial capacity predictions align closely with experimental observations, reducing errors from over 10% to less than 1%.

To incorporate strain decay, modifications are proposed for both the ACI 318 [22] and Eurocode 2 [23] equations:

The ACI 318 equation is adjusted from the modified ACI 318 Equation (1), and the final adjusted ACI 318 with the inclusion of strain decay factor ${(\eta }_{decay}$) is:

(5) $$P_{n}={(\eta }_{decay})\left(0.85\;f_{c\;}^{\prime }A_{g}){+(A}_{s}f_{y}\right)$$

The localized strain factor (ψ) is a correction factor introduced into the ACI 318 equation to account for the effects of local strain variations in concrete columns, as measured by fiber optic sensors (FOS). This factor modifies the conventional stress–strain relationship and column strength predictions by incorporating real-time, localized strain decay data ($\varepsilon_{decay}$) while also considering the cumulative effects of microcracking and material deterioration over time, and it can be expressed as:

(6) $$\eta_{decay}=1-k\frac{\varepsilon_{decay}}{\varepsilon_{yield}}$$

where $\varepsilon_{yield}$ is the strain of the concrete or reinforcement, serving as a reference limit; k is the calibration factor (empirically determined) to scale the impact of strain decay on the overall strength reduction; and $\varepsilon_{decay}$ represents the accumulated strain due to deterioration mechanisms (e.g., microcracking, creep, shrinkage) and can be modeled as a time-dependent parameter. An example of strain decay calculation is shown in Appendix A.

(7) $$\varepsilon_{decay}\left(t\right)=\varepsilon_{creep}\left(t\right)+\varepsilon_{shrinkage}\left(t\right)+\varepsilon_{microcracking}(t)$$

where $\varepsilon_{creep}\left(t\right)$ is the strain due to creep over time *t*, $\varepsilon_{shrinkage}\left(t\right)$ is the strain due to shrinkage effects, and $\varepsilon_{microcracking}(t)$ is the strain from progressive microcracking.

For slender columns, slenderness effects are combined with strain decay, as shown in Equation (6):

(8) $$P_{n}=\emptyset \left(0.85\;f_{c\;}^{\prime }A_{g+}A_{s}f_{y}\right)\times \;\;\frac{1}{1-\frac{Pu∆}{EI}}\times {(\eta }_{decay})$$

From the modified Eurocode 2 equation, the base Eurocode 2 equation was modified with the strain decay, and Equation (3) can be rewritten as Equation (9):

(9) $$N_{Rd}=\left[{(\eta }_{decay}\right)\times \alpha \times A_{C}f_{cd}+A_{s}f_{yd}]$$

For slender columns, considering critical load and strain decay, Equation (8) can be written as Equation (9):

(10) $$N_{Rd}=\left[{(\eta }_{decay}\right)\times \alpha \times A_{C}f_{cd}+A_{s}f_{yd}\times \;\frac{1}{1+\frac{N_{Ed}}{N_{cr}}\;}]$$

where $N_{Ed}$ is the applied load and $N_{cr}$ is the critical buckling load.

The proposed modifications address critical gaps in existing design codes, making them more applicable to low-strength and slender RC columns. By incorporating strain decay, the predictive accuracy of both ACI 318 and Eurocode 2 is enhanced, ensuring safer and more economical designs. Note that the value of *k* in Equation (6) is the calibration factor that adjusts the impact of the time-dependent deterioration factor ($\eta_{decay}$), represented by strain decay ($\varepsilon_{decay}$), on the overall strength reduction in RC columns. The value of *k* is determined empirically based on experimental observations and numerical analyses. The specific *k* value for this study is calibrated using strain data from BOTDA fiber optic sensors, ensuring that the proposed modified equations align with experimental and finite element analysis (FEA) results. The proposed approach can be adapted for real-time structural health monitoring by continuously integrating real-time strain data with predictive models that account for time-dependent effects like creep, shrinkage, and material degradation.

### 5.4. Comparison of the Modified Code Equations and Experimental Values

As discussed in Section 5.3, this research has proposed two modified equations from ACI 318 and Eurocode 2. The existing and modified equations for the load from ACI 318 [22] and Eurocode 2 [23] predictions were compared with the experimental values. From the proposed equations, “*k*” is the value of the calibration factor, which is the key parameter for the predictions from Equations (5) and (8), and it was optimized in this study. The “*k*” value is used in the proposed modifications to account for the sensitivity of the strain decay factor ${(\eta }_{decay})\;$ to deterioration mechanisms such as creep, shrinkage, and microcracking. It scales the effect of the strain decay ${(\eta }_{decay})$ relative to the reference yield strain $(\frac{\varepsilon_{decay}}{\varepsilon_{yield}}$). A typical “*k*” value in practice is considered to be between 0.10 and 0.20 for mild and severe environmental conditions, respectively. The value of “k” depends on the concrete strength, exposure conditions, structural usage, etc. This research evaluated the k = 0.10 value through empirical calibration based on the experiment and simulations. For this research, an optimized value of k = 0.10 was used to minimize prediction errors, especially under long-term loading conditions lasting up to 720 days. Table 5 illustrates a comparison of the experimental and predicted load values of ACI 318 [22] and Eurocode 2 [23].

**Figure 19:**
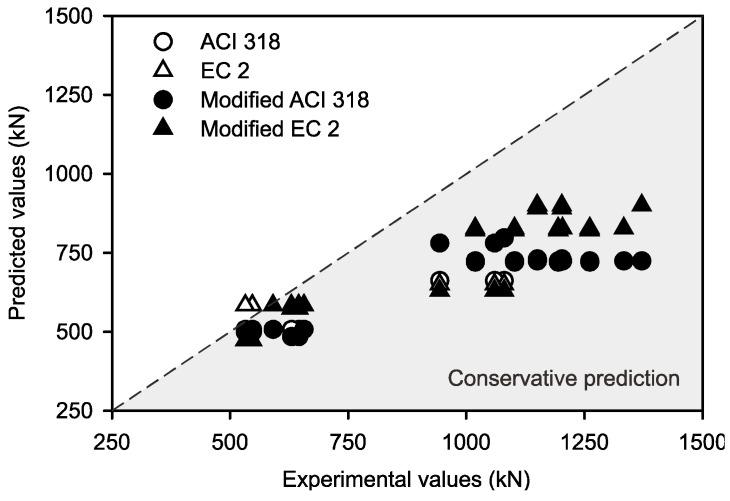
Comparison of experimental column strength values and different code equations.

The comparison was made between ACI 318 [22] and Eurocode 2 [23] load prediction values to study the predictions. Also, the modified equation predictions from the ACI 318 as well as Eurocode 2 equations were aligned with the ultimate load values from the experimental data. It was concluded that the ratio between the experimental and ACI 318 [22] and Eurocode 2 [23] load values were above 1.0 by considering the optimized “k” value as 0.1 for mild environmental conditions. Figure 19 shows the ratio of experimental and predicted values calculated from ACI 318 and Eurocode 2 with the existing and proposed equations. From the figure, it can be concluded that the ratios between the experimental and predicted values from the proposed equations through the ACI 318 [22] and Eurocode 2 [23] were improved by up to 5%, and can conservatively and accurately predict the service lives of the tested RC columns with exposure to mild environmental conditions over the time period.

## 6. Summary and Conclusions

The use of strain decay in axial capacity calculations markedly improves the accuracy of predictions for low-strength RC columns intended for long-term testing (2 years). Its integration into the ACI 318 and Eurocode 2 equations, supported by advanced monitoring technologies like BOTDA FOS, represents a significant advancement in structural design and assessment practices. The following conclusions can be drawn from this study:

- From the experimental investigations, the load values at the cracking, yield, and ultimate points decreased with the increase in the number of days for all series of all columns, which implied that the deformation plays a vital role over time in terms of environmental, creep, shrinkage, and microcrack factors.
- This experimental study evaluated the strain’s variational behavior through the conventional strain gauges and BOTDA FOS, which can be used for evaluation of the strain decay factor to improve the service life predictions of RC columns.
- Finite element analyses (Abaqus) were performed to predict the experimental column damage using the CDP model, which also proves that the experimental and validation values were in good agreement.
- η_decay_ was introduced into model strain reduction from creep and shrinkage, enhancing prediction accuracy by 3% for long-term performance.
- The ultimate load values were also predicted for all the tested columns through the ACI 318 and Eurocode 2 guidelines to ensure that the experimental values conservatively predicted values for specimens tested at t = 720 days.
- Findings were validated through experiments and simulations, ensuring 5% improvements in reliability for durable and sustainable existing low-strength RC columns.

## Figures and Tables

**Table 1:** Details of tested columns.

Series	Specimen	*b* (mm)	*H* (mm)	λ	*f’c* (MPa)	*ρ_ϕ_* (%)	Shear Reinforcement	FOS	Load Test (*t*), Days
I	C1-150	200 × 200	1500	26.0	24	2.0%	ϕ6 mm @ 100	A	28
	C2-150	200 × 200	1500	26.0	24	2.0%	ϕ6 mm @ 100	A	360
	C3-150	200 × 200	1500	26.0	24	2.0%	ϕ6 mm @ 100	A	720
II	C4-150	200 × 200	1500	26.0	24	2.0%	ϕ6 mm @ 100	B	28
	C5-150	200 × 200	1500	26.0	24	2.0%	ϕ6 mm @ 100	B	360
	C6-150	200 × 200	1500	26.0	24	2.0%	ϕ6 mm @ 100	B	720
III	C7-150	200 × 200	1500	26.0	24	2.0%	ϕ6 mm @ 100	C	28
	C8-150	200 × 200	1500	26.0	24	2.0%	ϕ6 mm @ 100	C	360
	C9-150	200 × 200	1500	26.0	24	2.0%	ϕ6 mm @ 100	C	720
IV	C10-170	200 × 200	1700	29.4	18	1.13%	ϕ6 mm @ 200	D	28
	C11-170	200 × 200	1700	29.4	18	1.13%	ϕ6 mm @ 200	D	360
	C12-170	200 × 200	1700	29.4	18	1.13%	ϕ6 mm @ 200	D	720
V	C13-180	200 × 200	1800	29.4	18	1.13%	ϕ6 mm @ 200	D	28
	C14-180	200 × 200	1800	29.4	18	1.13%	ϕ6 mm @ 200	D	360
	C15-180	200 × 200	1800	29.4	18	1.13%	ϕ6 mm @ 200	D	720
VI	C16-180	150 × 150	1800	41.6	40	1.13%	ϕ6 mm @ 150	D	28
	C17-180	150 × 150	1800	41.6	40	1.13%	ϕ6 mm @ 150	D	360
	C18-180	150 × 150	1800	41.6	40	1.13%	ϕ6 mm @ 150	D	720

Note: FOS is fiber optic sensor, *H* is height of the column, *b* is cross-section of the column, λ is slenderness ratio, *f’c* is compressive strength, *ρ_ϕf_* is reinforcement ratio, Type A is attached to the main reinforcement, Type B is attached to the shear reinforcement, Type C is attached to the surface and Type D is attached to the groove, and t is the testing duration.

**Table 2:** Mix proportions of concrete per cubic meter.

Mixture ID	CEM I (kg)	Fine Aggegates (kg)	Coarse Aggegates (kg)	Water + SP (kg)	Slump (mm)
M18	284	698	1257	173	95
M24	305	740	1140	165	90
M40	370	792	1084	157	85

**Table 3:** Mechanical properties of the concrete used in the experimental work.

Mixture ID	$\mathrm{Compressive}\;\mathrm{Strength}\;\mathrm{of}\;\mathrm{Cylinder}\;(f_{c}^{\prime })$		$\mathrm{Tensile}\;\mathrm{Strength}\;\mathrm{of}\;\mathrm{Cylinder}\;(f_{t}^{\prime })$		Modulus of Rupture $\left(f_{b}^{\prime }\right)$	
	Mean (MPa)	SD (MPa)	Mean (MPa)	SD (MPa)	Mean (MPa)	SD (MPa)
M18	19.1	2.1	2.1	2.2	2.8	2.6
M24	24.5	2.3	3.8	2.6	4.2	3.1
M40	42.2	3.2	4.2	4.1	5.4	4.2

**Table 4:** Experimental test results and performance indices of RC columns.

ID	*P_cr_*	Δ*_cr_*	*P_y_*	*P_u_*	Δ*_max_*	Δ*_y_* (mm)	Δ*_f_* (mm)	*µ_u_*	*µ_f_*	*S_e_*	*ζ*
	(kN)	(mm)	(kN)	(kN)	(mm)					(kN/mm)	(kN.mm)
C1-150	590.64	0.33	961.24	1215.61	3.17	0.64	6.71	4.95	0.10	1789.82	7800
C2-150	566.10	0.29	910.65	1104.26	2.75	0.29	3.58	9.48	0.08	8087.14	3950
C3-150	529.37	0.26	891.93	1006.59	2.31	0.71	4.01	3.25	0.18	1459.92	4250
C4-150	573.03	0.35	965.43	1187.53	2.93	0.62	5.23	4.73	0.12	2072.39	4350
C5-150	469.37	0.29	925.32	1094.38	2.55	0.10	5.48	25.50	0.02	18,979.00	5900
C6-150	374.67	0.26	893.67	984.21	3.00	0.75	5.08	4.00	0.15	1040.75	4200
C7-150	599.55	0.43	984.26	1203.45	2.51	1.50	5.8	1.67	0.26	1131.23	4550
C8-150	468.13	0.38	916.12	1094.53	4.21	1.32	5.71	3.19	0.23	968.76	4400
C9-150	467.73	0.27	853.56	959.28	3.92	0.31	5.86	12.65	0.05	3339.59	5950
C10-170	549.52	0.73	846.46	1053.12	1.83	1.75	1.95	1.05	0.90	436.90	560
C11-170	445.26	0.61	814.28	946.26	3.56	3.33	3.87	1.07	0.86	729.93	850
C12-170	352.38	0.56	791.26	884.35	3.58	2.31	3.21	1.55	0.72	677.98	1050
C13-180	593.49	0.35	891.59	989.25	3.77	2.78	3.95	1.36	0.70	3289.93	1280
C14-180	514.87	0.28	869.53	884.63	2.33	2.26	3.68	1.17	0.54	1838.82	1550
C15-180	451.26	0.24	842.58	865.38	4.50	4.42	5.89	1.13	0.68	337.79	2230
C16-180	521.93	0.32	795.86	1079.65	1.06	0.64	1.15	1.66	0.56	1631.03	360
C17-180	483.57	0.27	761.24	942.51	1.52	1.54	1.55	0.99	0.99	711.13	700
C18-180	416.39	0.25	749.19	892.47	1.68	1.86	1.49	0.90	1.24	615.68	790

Note: *P_cr_* is cracking load, Δ*_cr_* is deflection at cracking load, *P_y_* is yield load, *P_u_* is ultimate load, Δ*_max_* is maximum deflection, Δ*_y_* is yield deflection, Δ*_f_* is failure deflection, *µ_u_* is ductility at ultimate load, *µ_f_* is ductility at failure load, *S_e_* is effective pre-yield stiffness, *ζ* is energy absorption.

**Table 5:** Comparisons of experimental column strength according to current codes and proposed modifications to the code equations.

Specimen ID	Experiment Value (E), kN	Predicted Column Strength (P), kN				Exp/ Equation (2)	Exp/ Equation (3)	Exp/ Equation (5)	Exp/ Equation (9)
		Equation (2)	Equation (3)	Equation (5)	Equation (9)				
C1-150	1333.0	723.9	827.1	723.9	827.1	1.84	1.61	1.84	1.61
C2-150	1261.0	723.9	827.1	720.1	820.5	1.74	1.52	1.75	1.54
C3-150	1102.0	723.9	827.1	720.1	820.5	1.52	1.33	1.53	1.34
C4-150	1371.0	723.9	900.0	723.9	900.0	1.89	1.52	1.89	1.52
C5-150	1202.0	723.9	900.0	730.0	890.0	1.66	1.34	1.65	1.35
C6-150	1150.0	723.9	900.0	730.0	890.0	1.59	1.28	1.58	1.29
C7-150	1203.5	723.9	827.1	723.9	827.1	1.66	1.46	1.66	1.46
C8-150	1194.5	723.9	827.1	720.0	820.1	1.65	1.44	1.66	1.46
C9-150	1019.0	723.9	827.1	720.0	820.1	1.41	1.23	1.42	1.24
C10-170	656.0	554.4	631.1	554.4	631.1	1.18	1.04	1.18	1.04
C11-170	645.0	554.4	631.1	531.8	620.0	1.16	1.02	1.21	1.04
C12-170	630.0	554.4	631.1	531.8	620.0	1.14	1.00	1.18	1.02
C13-180	591.0	554.4	631.1	554.4	631.1	1.07	0.94	1.07	0.94
C14-180	547.0	554.4	631.1	546.2	520.0	0.99	0.87	1.00	1.05
C15-180	532.0	554.4	631.1	546.2	520.0	0.96	0.84	0.97	1.02
C16-180	1079.7	661.5	649.6	797.0	630.0	1.63	1.66	1.35	1.71
C17-180	1059.5	661.5	649.6	780.1	630.0	1.60	1.63	1.36	1.68
C18-180	943.6	661.5	649.6	780.1	630.0	1.43	1.45	1.21	1.50
Mean (E/P)						1.45	1.32	1.42	1.29
STD.						0.30	0.25	0.29	0.27

**Note:** The calibration factors k = 0 and 0.10 were adopted and used in the calculations for specimens tested at 28 days and more than 28 days (270 and 360 days), respectively.

## Data Availability

The details of the test data will be made available on request.

## Appendix A

Example of strain decay calculations from Equation (A1):

(A1) $$\eta_{decay}=\;1\;-\;k\frac{\varepsilon_{decay}}{\varepsilon_{yield}}$$

where $\varepsilon_{yield}\;$ is the yield strain of the concrete or reinforcement, serving as a reference limit; *k* is the calibration factor (empirically determined) to scale the impact of strain decay on the overall strength reduction; and $\varepsilon_{decay}$ represents the accumulated strain due to deterioration mechanisms (e.g., microcracking, creep, shrinkage) and can be modeled as a time-dependent parameter. For the calculation of $\eta_{decay}$, decay strain and yield values were taken from the height vs. strain data of the experimental column (Figure 9).

For column (C1-150), the experimental ultimate load value was obtained as 1333 kN. The predicted values from Equations (2), (3), (5), and (9) are shown in Table 5 for the same column. $\varepsilon_{yield}$ = 0.00356 and $\varepsilon_{decay}$ = 0.005760 for column (C1-150). The optimized “k” value of 0.1 for mild environmental conditions was considered. The $\eta_{decay}$ can be calculated as:

$$\eta_{decay}=0.8382$$
